# A workshop considering genetic research and data collection with American Indian and Alaska Native people outside of Tribal jurisdiction

**DOI:** 10.3389/fgene.2026.1802493

**Published:** 2026-08-31

**Authors:** Julie A. Beans, Jessica W. Blanchard, Vanessa Y. Hiratsuka, Taiawagi Helton, Stephanie Russo Carroll, Denise A. Dillard, Raymond Orr, Francine C. Gachupin, Sara C. Hull, Rodney C. Haring, Nanibaa’ A. Garrison, Bobby Saunkeah, Krystal S. Tsosie, Aaron J. Goldenberg, David Wilson, Richard R. Sharp, Joseph Yracheta, Erika Blacksher, R. Brian Woodbury, Dalaki Livingston, Paul Spicer

**Affiliations:** 1 Research Department, Southcentral Foundation, Anchorage, AK, United States; 2 Center for Applied Social Research, University of Oklahoma, Norman, OK, United States; 3 College of Law, University of Oklahoma, Norman, OK, United States; 4 Native Nations Institute at the Udall Center for Studies in Public Policy, University of Arizona, Tucson, AZ, United States; 5 Mel and Enid Zuckerman College of Public Health, University of Arizona, Tucson, AZ, United States; 6 Institute for Research and Education to Advance Community Health, Elson S Floyd College of Medicine, Washington State University, Spokane, WA, United States; 7 Department of Political Science, University of California, Santa Barbara, CA, United States; 8 Department of Family and Community Medicine, College of Medicine, University of Arizona, Tucson, AZ, United States; 9 National Human Genome Research Institute, National Institutes of Health, Bethesda, MD, United States; 10 Center for Indigenous Cancer Research, Roswell Park Comprehensive Cancer Center, Buffalo, NY, United States; 11 Institute for Society and Genetics, University of California, Los Angeles, CA, United States; 12 Institute for Precision Health, David Geffen School of Medicine, University of California, Los Angeles, CA, United States; 13 Division of General Internal Medicine and Health Services Research, David Geffen School of Medicine, University of California, Los Angeles, CA, United States; 14 Department of Health, Chickasaw Nation, Ada, OK, United States; 15 School of Life Sciences, Arizona State University, Tempe, AZ, United States; 16 Department of Bioethics, Center for Community Health and Genomic Equity, Case Western Reserve University School of Medicine, Cleveland, OH, United States; 17 Indigenous Health, School of Medicine and Health Sciences, University of North Dakota, Grand Forks, ND, United States; 18 Biomedical Ethics Research Program, Mayo Clinic, Rochester, MN, United States; 19 Native BioData Consortium, Eagle Butte, SD, United States; 20 Johns Hopkins Bloomberg School of Public Health, Baltimore, MD, United States; 21 Department of History and Philosophy of Medicine, University of Kansas Medical Center, Kansas City, MO, United States; 22 Department of Communication, University of Utah, Salt LakeCity, UT, United States

**Keywords:** Alaska Native, American Indian, genetic research, genomics, Tribal sovereignty, urban

## Abstract

**Introduction:**

In the United States (US), Tribes are sovereign nations and have the right to oversee research conducted with Tribal citizens. However, it is unclear who should approve research protocols when data from American Indian and Alaska Native (AIAN) people are collected off Tribal lands. As genetic research continues to advance and transform the delivery of healthcare, equitable inclusion of AIAN people is necessary, but oversight of research needs clarity.

**Methods:**

We held a 3-day workshop with US thought leaders on genetic and other health research with AIAN people in urban areas to explore views and values on this issue and to discuss potential policy and practice solutions.

**Results:**

Thirty-six individuals attended. Solidarity surfaced as a foundational motivation for Tribal Nations to review research conducted with AIAN people, whether on Tribal lands or not. Understanding data from Indigenous perspectives was identified as a way to ensure appropriate AIAN community protections are in place. Three discrete areas to improve policy were suggested–Tribal, Academic Institution, and National–to protect AIAN people participating in research both on and off Tribal lands.

**Discussion:**

Researchers, whether Indigenous or not, must recognize Tribal sovereignty and operate in solidarity with the applicable and most appropriate ethical principles and regulations.

## 1 Introduction

In the United States (US), Tribal Nations have the inherent right to self-determination expressed as self-governance. Tribal sovereignty is defined as the ability to exercise authority by groups of culturally distinct peoples with recognizable governments, and often a defined territory predating European contact (Harding et al., 2012). The right to regulate activities within Tribal jurisdiction and with Tribal citizens is therefore an essential responsibility–including the right to regulate research. Many Tribal Nations institute research review processes through research codes, established practices and procedures, Tribal Institutional Review Boards, and community-level ethics reviews (Around Him et al., 2019; Hiratsuka et al., 2017; Carroll et al., 2022). Research oversight within Tribal jurisdictions can be well defined, but the process for extending protections to Tribal citizens outside of jurisdictional boundaries is less clear. Some Tribes further define their responsibility to protect their citizens beyond their lands and extend expectations for ethical research beyond their federally-defined Tribal jurisdiction (Carroll et al., 2022). Despite this ambiguity, Tribes across the US are clear on the need to be included in planning, collecting, analyzing and disseminating research studies conducted with their citizens (Tribal Collaboration Working Group, 2018; Garba et al., 2023; Trinidad et al., 2022).

In 2023, there were about two million American Indian and Alaska Native (AIAN) people enrolled across 574 federally recognized Tribes in the US. (Murray, 2023) Geography and proximity to Tribal lands is not a requirement for Tribal enrollment or AIAN identity (Haozous et al., 2014). Federal policies (e.g., Termination Era, Indian Relocation) (Burt, 1986; Deloria, 1976) intentionally removed AIAN people from Tribal lands and Tribal jurisdiction protections, and into urban locations as a means of diluting communal rights to land and natural resources and to dissolve familial cultural connections. Today, about 29% of Tribal citizens reside on Tribal lands, with about 71% residing outside of Tribal lands, including in urban areas (UIH, 2026) The lasting negative impact of these acculturation policies are seen in both the health and socio-economic status of AIAN people (Walls and Whitbeck, 2012), and there is a continued lack of community protections for Tribal citizens residing outside of federally defined Tribal geographic jurisdictions who participate in research (Beans et al., 2019). Concerns regarding the potential misuse of biospecimens and data, exploitation, misrepresentation, and the perpetuation of stigmatizing narratives through research are central for AIAN people regardless of residence (Garrison and Carroll, 2023; Haozous et al., 2021).

Cultural, ethical, and legal issues are important to consider as genetic research advances and transforms healthcare delivery. Participation by AIAN collectives and individuals in genetic research is seen by some as particularly important given the historical lack of diversity in genetic research and the resulting potential irrelevance of innovations to diverse populations served by healthcare systems (Hindorff et al., 2018). Large research efforts such as the National Institutes of Health’s *All of Us* Research Program, which recruits participants with the goal of building a participant database representative of the diversity of the US, raises urgent questions about data collection among AIAN populations outside of Tribal jurisdictions. Whether intentional or not, some recruitment efforts target AIAN people outside of Tribal lands, circumventing procedures for Tribal oversight of data and research that often include secondary use of data (Haozous et al., 2021; Tsosie et al., 2019). US human subjects research guidelines insufficiently address the needs and concerns of Tribal communities and align to non-Indigenous ethical norms that do not address Tribal sovereignty, group risks, and group harms. Absent of alignment to community-centered research policies, investigator projects funded by US federal government may inadequately protect Tribal citizens residing outside of federally defined Tribal jurisdiction.

The Center for the Ethics of Indigenous Genomic Research (CEIGR) is a multidisciplinary consortium of community-placed Tribal partners, university researchers, cancer center researchers, and community-based institutions working collaboratively to conduct research on the ethical, legal, and social implications (ELSI) of genomic research in AIAN communities in the US. (Hiratsuka et al., 2020) CEIGR emerged in response to critical and unresolved issues resulting from unethical genomics research practices within AIAN populations. Genomics has thus far mostly failed to produce clinical and other measurable benefits for AIAN communities, so hesitations about participating in genetic research persist (Caron et al., 2020). While the decision not to engage in genomic research may be the best decision for some Tribes, the CEIGR Consortium also recognizes that some AIAN communities may wish to participate in research. CEIGR promotes and encourages serious and sustained engagement with AIAN communities about the role of genomic research in their communities and is committed to addressing unresolved ELSI questions.

CEIGR organized a workshop to explore current and emerging cultural, ethical, and legal issues about genomic research with Tribal citizens, including biospecimen collection, occurring outside of Tribal jurisdictions. Here, we describe and discuss the themes identified in the workshop which explore the roles and responsibilities of Tribal citizens, Tribes, investigators, research institutions, Federal agencies, and other relevant entities with respect to the conduct and governance of genetic research occurring with Tribal citizens outside of Tribal jurisdictions.

## 2 Materials and methods

We conducted a 3-day virtual workshop in spring 2021 to facilitate discussion on the collection of AIAN genetic data at locations outside of federally defined Tribal jurisdictions. We brought together individuals with expertise in working with AIAN communities, federal research policies, genetics research, healthcare, and ELSI research to identify experiences, concerns, and suggestions about genomic data collection occurring outside of federally defined Tribal jurisdiction, such as in urban settings.

### 2.1 Workshop design

A subset of CEIGR consortium members planned and conducted the workshop (CEIGR planning group), with input from the entire consortium. Each 3-h workshop session opened with remarks and relevant background, then moved into a facilitated discussion (Table 1). CEIGR planning group members took notes on main points of the conversation. Written informed consent from the participants was not required to participate in this workshop discussion for the publication of personal statements in accordance with the national legislation and the institutional requirements. This workshop was designed to facilitate conversation and all participants agreed to recording and transcription of each session for the purposes of manuscript development.

**TABLE 1:** Workshop information.

​	Date	Emphasis	Presenter
Day 1	5 March 2021	Legal constructs and foundational concepts (Tribal citizenship, Tribal jurisdiction, genetic research, Tribal governments)	Taiawagi Helton JD ‐ American Indian and Indigenous Peoples Law Professor, University of Oklahoma
Day 2	19 March 2021	Genomic research: Past examples/Responses, urban Indian contexts	Abigail Echo-Hawk (Pawnee) MA ‐ Director, Urban Indian Health Institute; Executive Vice President, Seattle Indian Health Board
Day 3	2 April 2021	Indigenous data sovereignty	Stephanie Carroll (Ahtna) DrPH, MPH ‐ Director, Collaboratory for Indigenous Data Governance, University of Arizona

The first session explored Tribal jurisdiction over research from ethical and legal points of view. This introduction facilitated an understanding of Tribal sovereignty. The second session began with introductory remarks about current research happening with AIAN people in urban areas, and approaches to Tribal oversight. The third session focused on the Indigenous Data Sovereignty movement, orienting attendees to data governance initiatives in Indigenous communities across the US and worldwide.

### 2.2 Attendees

Invitations to attend the workshop were extended to members of the CEIGR consortium, Federal research agency employees, and some individuals with no prior engagement with the CEIGR but with relevant subject-matter expertise. Attendance was sought from AIAN community members, Tribal employees, US National Cancer Institute designated cancer center researchers, university and industry researchers, clinicians, and Federal government representatives. Thirty-six individuals attended at least one workshop day. Attendance varied across each day, though most were present for all workshop days. Attendees had experience working with AIAN communities in areas of bioethics, genetics, community engagement, public policy, health disparities, biobanking and law.

Following each session, the CEIGR planning group performed rapid content analysis (Vindrola-Padros and Johnson, 2020) of planning group notes taken during the workshop to generate summary reports. Notes were cross-checked against video recordings and transcripts as needed for accuracy. Summary reports were circulated to all meeting attendees, who were asked to confirm that each report captured the main discussion points.

Following the initial summary reports, a second review of workshop notes and transcripts was conducted to identify salient themes, categorize themes according to workshop topics, and identify thematic patterns.

## 3 Results

The goal of this workshop was to consider AIAN genetic data collection outside of federally defined Tribal jurisdictions, such as in urban areas. Attendees expressed the need for researchers to understand the nature of relationships required to conduct meaningful health research broadly and genomic research specifically. Workshop attendees identified a range of current and emerging bioethical issues—in both basic and clinical research—and shared a broad range of perspectives. While the discussion focused on legal concepts and terminology pertaining to Tribal jurisdiction, workshop attendees were clear that research occurring outside of federally defined Tribal jurisdictions must be guided by a broader set of ethical and relational principles that are foundational for legal and policy frameworks.

The importance of community-based and Tribally driven research resurfaced throughout the workshop. In addition, attendees described the essential need for strong relationships between researchers, funders, and AIAN communities to foster equitable participatory research projects. Community-based research with equitable relationships is not unique to Tribal contexts but was emphasized as vital to genomic research with AIAN populations and individuals by all attendees. For example, one attendee shared, *“…even if they [outside researchers] are willing to do the work of forming relationships [it] does not mean they’re ready or not likely to inflict harm,”* (Attendee 12) highlighting the nuance involved in forming bi-directional working relationships with Tribal communities and the time investment required to build trust.

### 3.1 Indigenous views of data

Attendees spoke to the importance of understanding that terms such as jurisdiction and Tribe among many others are often rooted in colonial understandings and definitions that prioritize individual rights, and from Indigenous perspectives, can be exclusionary of group or community rights. Individual rights were not seen as superseding collective harms, and research guidelines must consider how individual participation may undermine Tribal sovereignty. The emphasis on individualism in Western philosophy serves the objectives of individual investigators rather than serving, or even harming, Indigenous Peoples. Attendees noted that standard research practices like informed consent, notions of risk, and exempting secondary data from Tribal oversight and regulation, reflect an *individual versus collective* framework and are therefore inadequate.

The discussion was clear that, from Indigenous perspectives, AIAN people have a responsibility to data, and these data have a responsibility to the people. This reciprocal relationship was described as extending beyond federal definitions of human subjects research. Within Western academic framing, there are often dichotomies with respect to data (e.g., human subjects vs. non-human-subjects, collective vs. individual, quantitative vs. qualitative), whereas some Indigenous perspectives were described as more holistic and perceive data as a being (Huaman, 2022). The notion of data as a being is a dynamic interactive understanding that data are to be treated with mutual respect because data are intertwined not just with the individual who provided those data, but with the Peoples. These conflicting definitions of data can and have led to miscommunication and misunderstandings, resulting in harm and ultimately distrust (Dillard et al., 2018). The differing definitions of data also impact who, when, where, why, and how respect for persons and respect for communities is seen and shown. Indigenous perspectives include a more fluid definition or view of what data are and in turn how data should be treated and handled. As one attendee noted, *“For those of us who are Indigenous Peoples working in this world, we forget that ancestral connection to it…. That is data, that it lives, it breathes, it speaks, it carves, sings, prays. And that we have a connection to it, to our ancestors, to the past, for the present, and always for the future. And that always has to be where our conversations around Indigenous data is grounded in.”* (Attendee 3).

### 3.2 Solidarity

Although the scope of the workshop was well-defined, the ethical principles that should inform AIAN data governance outside of Tribal lands remain unclear. A guiding principle of CEIGR is respect for Tribal sovereignty in the conduct of genomic research (Hiratsuka et al., 2020; Blanchard et al., 2020), but solidarity was a key principle that surfaced the first day of the workshop. Solidarity was described as a key ethical principle in protecting Tribal resources, including genetic data, that supplements sovereignty and underpins the necessity of Tribes protecting their citizens (Saunkeah et al., 2021), whether they live on or off of Tribal lands. As one attendee explained, *“Solidarity means caring for members of the group. This perspective on a Tribal community’s obligations to its own members should be communicated to researchers.”* (Attendee 1).

Attendees recognized that communicating solidarity as a principle to researchers was vital. Solidarity emerged as an important underpinning of the responsibility to protect AIAN people and was described, not only as a responsibility Indigenous investigators have to data collected from AIAN Peoples and individuals, but also as a responsibility that data (collected from AIAN people) has to AIAN people, inclusive of biospecimens and genomic data. Attendees indicated that this responsibility is to protect the larger group, and that the participation of only a few individuals could compromise the groups’ information. Attendees noted that group protections should be extended beyond federally defined definitions of AIAN people to all Indigenous Peoples in the US regardless of federal recognition. It was emphasized that protecting the larger group required work across national borders since genetic inferences can be drawn across groups with shared histories and enduring social and familial ties. The principle of solidarity encompasses protection from harm for all these groups and is based on a concept of relationality much broader than biology alone, particularly when considered from Indigenous perspectives. One attendee articulated, *“…the difference in how people looked at relationality in a cultural way versus [the] idea of genetic relationality can be exclusionary, or it can be inclusive, right? And so the idea to go beyond borders and to explore that it is an inclusive endeavor for Indigenous Peoples with respect to, for example, data governance … it also requires us to rethink the ethics that go with that.”* (Attendee 2).

Thus, AIAN data are inextricably linked to a set of values that bind individual Tribal citizens to a larger set of relationships and responsibilities. Solidarity was described as not just a tension between individual and collective protections—this dichotomy was viewed as limiting—rather, the principle of solidarity encompasses an overarching set of inherent obligations and responsibilities not bound by jurisdictions or borders.

### 3.3 Misaligned values and inadequate protections

Solidarity was recognized as an ethical principle aligned with many Tribal communities’ values but may not be shared more broadly across the US. Although, solidarity may be a relevant and integral ethical framework applied in many Tribal settings (Saunkeah et al., 2021), it directly conflicts with democratic principles that many US citizens uphold, which emphasize individuals’ liberties over group rights. One attendee highlighted this point by stating, *“We [Americans] seem perfectly comfortable recognizing corporate rights as a form of collective right, but real discomfort with Tribal rights.”* (Attendee 6).

### 3.4 Regulatory gaps

Attendees acknowledged that federal regulatory boards were developed to protect the individual and were described as providing inadequate protection and consideration for the group, whether that be Tribes or Tribal citizens as individuals where they live. “…*they [IRBs] decide decisions that do not take into consideration the true ethical concerns that would come from an Indigenous perspective … so we also have to recognize that these boards, again, were never made to benefit Indigenous Peoples.”* (Attendee 3) An example brought forward by some attendees was the Belmont Report’s failure to address group harm (Hull and Wilson, 2017), as well as protections when considering informed consent for genomic data, which can be re-identifiable at the group level (Tsosie et al., 2019). Without these considerations being communicated during the informed consent process, attendees questioned if true informed consent is possible.

For these reasons, among others, investigators should begin conversations and planning well before genomic data collection with AIAN people begins, whether on or off Tribal lands. *“[W]hen we think about community engagement, US falls silent on engaging communities before samples are collected or respecting cultural significance, rights, interests, getting approval to do secondary analyses … ”* (Attendee 9) Planning discussions should include how data are being collected, the types of data being collected, who is managing those data, and what type of agreements need to be in place for analysis and long-term storage of those data–including biospecimens and genomic data. Oftentimes, preliminary discussions between AIAN communities and investigators, biobanks, and institutions about inclusion in genomic research focus on *how* to recruit and consent AIAN individuals, but attendees instead urged the discussion to start with what types of data are being collected and what happens to those data to fully respect Tribal data sovereignty within the US context.

### 3.5 Tribal community protections in research

Attendees noted that conversations about Tribal data governance have been occurring for decades with the same concerns repeatedly expressed by Tribes (Parran et al., 1954). Thus, attendees indicated that formal policy changes are necessary to advance the conversation and that policy should be co-created with Tribes, institutions, and federal funding agencies (Parran et al., 1954; Pokagon, 1893; Cochran et al., 2008; Caldwell et al., 2005). Attendees also noted that the policies must include group-level protections for AIAN communities by integrating relevant cultural values when considering equitable inclusion of AIAN Peoples in genomic research moving forward.

### 3.6 Tribal focus

Strengthening community protections and decreasing risks of group harm to Tribes would require a broader application of what necessitates regulatory review than the current US federal regulatory requirements for research (Haring et al., 2021). Attendees recommended developing models of Memorandum of Understanding language grounded in rights afforded to AIAN people by treaties, that would legally justify oversight by urban organizations that serve AIAN people or community advisory boards for research in areas outside of Tribal jurisdiction. Additionally, attendees urged improved provenance tracking as data would become increasingly disconnected from any people or group with repeated sharing and reuse. Provenance tracking is labeling the origins of data, including Indigenous lands, knowledge, and resources (Golan et al., 2022). In addition to provenance tracking, attendees recommended uses of metadata which specifies permissions. Attendees also encouraged Tribal leadership to develop recommendations and offer guidance about participation in a study. *“I really like the idea of going before Tribal councils and getting their official decision or position on a research study. And then be able to communicate that out to the urban population, to its urban members and let them make that informed decision on whether to participate or not.”* (Attendee 10).

### 3.7 Academic institution focus

Attendees viewed changes to the organizational ethics of institutions and strengthening institutional responsibilities to oversee individual researchers as necessary to protect AIAN people. Fostering relationships between academic institutions, such as establishing academic institutional Tribal research policies or guidelines for their research community and Tribes was put forth as an opportunity to instill AIAN community protections at the institutional level. One example offered was to include dropdown menus on regulatory forms that include only those Tribes with whom the academic institution has established agreements in place as those with whom investigators are permitted to collaborate. In instances where the Tribe is not listed on the dropdown menu, a community engagement plan would be required. Another example was for institutions such as Universities to create and enforce standards for its investigators to abide by when conducting work with AIAN people and groups, such as those set forth in the University of Arizona’s “Native American or International Indigenous Populations in Human Research” guiding document (Marley, 2019).

### 3.8 Federal focus

Attendees discussed tangible steps that could be taken at the national level, such as advocating for funding mechanisms that prioritize Tribal oversight and direction like the Native American Research Centers for Health. Data trusts were also discussed as a promising model of collaborative governance. Data trusts could be governed by a board with responsibility for overseeing data usage and protecting participants. Attendees also recommended that urban confer (Parker, 2020; UCP, 2026) be integrated into the research process, to supplement Tribal consultation. Currently, only the Indian Health Service Urban Confer Policy offers guidance for those seeking review by Urban Indian Organization leadership on issues related to health of urban AIAN people under the Indian Healthcare Improvement Act (UCP, 2026).

Through discussion, the US federal government was viewed as responsible for federally funded research. Attendees indicated that the National Institutes of Health should oversee investigators receiving funds and intervene when data are collected without Tribal approval. *“I do not know what IRBs [Institutional Review Boards] need to hear, but I liken it to not being able to just go to another country and take their data. Tribal citizens are citizens of US and their own Tribal Nations. You cannot just go off the reservation and take people’s samples. … IRBs are not the regulatory authority, they are a review authority. NIH [National Institutes of Health in US] is [the] regulatory authority.”* (Attendee 8) Along similar lines, attendees questioned the role of the federal Trust Responsibility in protecting Tribal resources of individual beneficiaries living outside of Tribal lands.

The US federal government has yet to reckon at a national level with the legacies of injustices US federal policy inflicted upon AIAN people. In contrast, the Canadian Government has begun to undertake the process required by translational justice for Indigenous Peoples via the Canadian Truth and Reconciliation Commission (Stanton, 2011; Justice, 2018), which was established by the Canadian Government as part of the class action settlement agreement where 14,903 Indian Residential School survivors filed claims against the Canadian government for the negative traumatic impacts the Indian Residential School policy had and continues to have on Indigenous Peoples of Canada (Stanton, 2011). This unfulfilled need for reconciliation in the US was described as an essential step needed to achieve equity for AIAN people on both Tribal and non-Tribal lands.

*“In Canada, there's been a movement of truth and reconciliation between the Canadian government and the First Nations, Inuit and Métis, three predominant Indigenous groups of Canada. In that … various forms of governance and reconciliation. Not perfect, but I think a start [is] that the US has not really bridged into some of the workings of many things including research. So, I think that looking at their model could provide some insight into the discussion that's going on as well. Because a lot of it is based on kind of historical injustice. And what that means, not only from a Canadian perspective, but from the perspective that we've all felt as Indigenous Peoples and the historical traumas and issues that we've faced over time.”* (Attendee 2).

Last, attendees encouraged the use of a broader ethical framing to avoid constructing a core ethical tension often inappropriately presented as “group versus individual” rights. Rather than referring to AIAN groups as vulnerable, the discussion emphasized movement towards clear ethical norms that honor group autonomy which could include a sovereignty and/or human rights framing within biomedical research.

## 4 Discussion

This workshop provided space to operationalize the complex ethical principles and practices that incorporate and prioritize AIAN Peoples and individuals’ experiences and responsibilities. Solidarity, a dimension of distributive justice (Reichlin, 2011), was described as a pillar of Tribes’ and Tribal citizens’ collective responsibilities to individual members and to AIAN Peoples and individuals data, inclusive of biospecimens. Collective responsibilities extend beyond citizens of a particular Tribe to include other Indigenous Peoples in the US, such as those with and without federal recognition, as well as Indigenous Peoples worldwide.

While Indigenous Data Sovereignty was addressed in the workshop, attendees mostly emphasized the unique context of federally recognized Tribes in the US. Supporting solidarity with AIAN populations in the US requires trusting relationships between investigators, institutions, funders, and Indigenous Peoples’ data. The misalignment between Indigenous and Western views is compounded by the chronic underfunding and, in many cases, inefficient structure of federal programs designed to support the health, social, and economic wellbeing of AIAN people (United States Commission on Civil Rights, 2018). Such underfunding is the lasting legacy of federal policy that perpetuates the reality of unmet basic needs and contributes to inequities experienced by AIAN people (United States Commission on Civil Rights, 2018).

Developing an ethical approach to include AIAN Peoples and individuals in genomic research consonant with understandings of relationship and responsibility will require considerable effort. Workshop attendees made clear that respect for Tribal sovereignty within the US is a necessary foundation for change as it relates to oversight of data from Tribal citizens. Tribal sovereignty in the US is largely defined in political and legal terms delineating a clear nation to nation relationship between Tribal Nations and the federal government, but it does not make clear the broader notion of relatedness found in human rights framing asserted by some Indigenous groups globally. In the context of research involving multiple sovereign entities in the US, including Tribal and federal agencies, there is an expectation for collaborative governance. Dialogue about ethics, across these very different intellectual traditions suggests intriguing possibilities for advancing dialogues not just in ethics, but also with respect to Indigenous science and futurity.

It is time to reexamine current institutional and federal policies to incorporate more meaningful attention to the views, values, and needs of Tribal communities. At the state and institutional levels example, the Arizona Board of Regents implemented the Tribal Consultation Policy 1–118 in 2018 to which both Arizona State University and the University of Arizona issued supplemental guidance specifying additional requirements for University investigators working with Tribes (Arizona TUo, 2023), thus putting in place stronger policies than the institutional example at the time the workshop took place (Marley, 2019). Such policies support Tribes in providing community protections for all Tribal citizens on and off of Tribal lands. At the federal level, the guidance issued by the National Institutes of Health in 2022 acknowledges the importance of Indigenous data sovereignty and aims to “facilitate respectful, sustained, mutually beneficial, and equitable partnerships between AIAN Tribes, researchers, and the biomedical research enterprise” (NIH Wash DC, 2022).

There are limitations to this workshop summary. Although Indigenous and non-Indigenous individuals were invited to attend the workshop, not all invitees were able to attend and workshop attendance varied across the 3 days, which may have influenced the workshop discussion.

Furthermore, the vital importance of relationships and connections for Indigenous Peoples is absent from widely available frameworks developed for academic audiences, inscribed within a language of individual rights. More work is needed to engage Tribal communities in conversations about research and regulatory requirements to enhance policy. Genomic data are considered a Tribal resource, that is held in a trust relationship by the Tribe and Tribal citizens. Researchers, whether Indigenous or not, are expected to recognize and operate under the values and governance structures defined by the Tribe or Tribes with whom the researcher is working, acting in solidarity with the applicable and most appropriate ethical principles and regulations.

## Data Availability

Requests for data supporting the conclusion of this article can be made to the corresponding author. Requests may required to be reviewed by involved Tribal Review Bodies as appropriate.
