# Correction: A workshop considering genetic research and data collection with American Indian and Alaska Native people outside of tribal jurisdiction

**DOI:** 10.3389/fgene.2026.1984784

**Published:** 2026-09-23

**Authors:** Julie A. Beans, Jessica Blanchard, Vanessa Hiratsuka, Taiawagi Helton, Stephanie Carroll, Denise Dillard, Raymond Orr, Francine C. Gachupin, Sara Hull, Rodney C. Haring, Nanibaa' A. Garrison, Bobby Saukeah, Krystal S. Tsosie, Aaron Goldenberg, David R. Wison, Richard R. Sharp, Joseph Yracheta, Erika Blacksher, Richard Brian Woodbury, Dalaki Livingston, Paul Spicer

**Affiliations:** 1 Southcentral Foundation, Anchorage, United States; 2 The University of Oklahoma, Norman, United States; 3 The University of Arizona, Tucson, United States; 4 Washington State University, Pullman, United States; 5 University of California Santa Barbara, Santa Barbara, United States; 6 National Institutes of Health, Bethesda, United States; 7 Roswell Park Comprehensive Cancer Center, Buffalo, United States; 8 University of California Los Angeles, Los Angeles, United States; 9 Chickasaw Nation Medical Center, Ada, United States; 10 Arizona State University, Tempe, United States; 11 Case Western Reserve University Cleveland Health Sciences Library, Cleveland, United States; 12 University of North Dakota, Grand Forks, United States; 13 Mayo Clinic Minnesota, Rochester, United States; 14 Johns Hopkins University, Baltimore, United States; 15 The University of Kansas Department of History and Philosophy of Medicine, Kansas City, United States; 16 University of Utah Health, Salt Lake City, United States

**Keywords:** Alaska Native, American Indian, genetic research, genomics, tribal sovereignty, urban

There was a mistake in Table 1 as published. To respectfully acknowledge and accurately document, names and title must be capitalized appropriately. The corrected Table 1 appears below.

**Table:** 

Day 1	5 March 2021	Legal constructs and foundational concepts (Tribal citizenship, Tribal jurisdiction, genetic research, Tribal governments)	Taiawagi Helton JD ‐ American Indian and Indigenous Peoples Law Professor, University of Oklahoma
Day 2	19 March 2021	Genomic research: Past examples/Responses, urban Indian contexts	Abigail Echo-Hawk (Pawnee) MA ‐ Director, Urban Indian Health Institute; Executive Vice President, Seattle Indian Health Board
Day 3	2 April 2021	Indigenous data sovereignty	Stephanie Carroll (Ahtna) DrPH, MPH ‐ Director, Collaboratory for Indigenous Data Governance, University of Arizona

The original version of this article has been updated.

